# Society for Cardiovascular Magnetic Resonance perspective on the 2021 AHA/ACC Chest Pain Guidelines

**DOI:** 10.1186/s12968-021-00835-z

**Published:** 2022-01-03

**Authors:** Andrew E. Arai, Raymond Y. Kwong, Michael Salerno, John P. Greenwood, Chiara Bucciarelli-Ducci

**Affiliations:** 1 Private Consultant, Kensington, MD 20895 USA; 2 Brigham and Women’s Hospital, Boston, MA USA; 3 Stanford University, Palo Alto, CA USA; 4 Leeds Institute of Cardiovascular and Metabolic Medicine, University of Leeds, Leeds, UK; 5 Royal Brompton and Harefield Hospitals, Guys’ and St Thomas NHS Hospitals and School of Biomedical Engineering & Imaging Sciences, King’s College London, London, UK

## Society for Cardiovascular Magnetic Resonance perspective on the 2021 AHA/ACC Chest Pain Guidelines

Diagnostic and treatment guidelines serve several important purposes with an overall aim to improve medical care. The 2021 American Heart Association (AHA)/American College of Cardiology (ACC) Chest Pain Guidelines [1, 2] represent a dramatic evolution from the prior 2012 ACC/AHA Chest Pain Guidelines [3]. For the practitioner that uses or performs cardiovascular magnetic resonance (CMR), the release of new guidelines is an opportunity to reassess what we do, how we do it, and how CMR should be used relative to other imaging modalities. Guidelines translate scientific evidence into recommendations on how to approach specific patient-related conditions. Though representing the “state-of-the-art” at the time of publication, guidelines ultimately represent the opinions of experts in the field and the quality of contemporaneous published literature. Inevitably, not all differences in opinion can be incorporated. The Society for Cardiovascular Magnetic Resonance (SCMR) endorsed the 2021 AHA/ACC Chest Pain Guidelines as they accurately incorporate 15 indications for CMR and capture a large proportion of what CMR has to offer patients and clinicians in the evaluation of acute and stable chest pain. This document aims to summarize the new 2021 AHA/ACC Chest Pain Guidelines from an SCMR perspective, to highlight the current role for CMR, to identify where knowledge gaps exist, and to describe areas where some CMR expert opinions may differ with the Guidelines. We hope this effort stimulates debate and more importantly stimulates research efforts to refine and expand appropriate CMR indications in future international guidelines.

## Indications for CMR in the 2021 AHA/ACC Chest Pain Guidelines

**Fig. 1:**
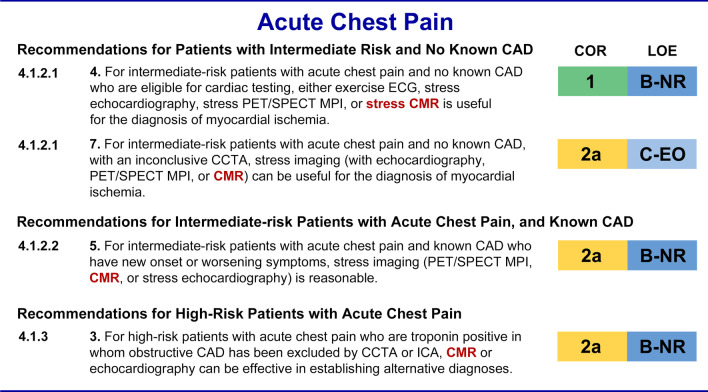
Acute Chest Pain Recommendations for CMR [1, 2]. *CAD* coronary artery disease, *CCTA* coronary artery computed tomography angiography, *CMR* cardiovascular magnetic resonance, *ECG* electrocardiogram, *ICA* invasive coronary angiography, *MPI* myocardial perfusion imaging, *PET* positron emission tomography, *SPECT* single photon emission computed tomography

The 2021 AHA/ACC Chest Pain Guidelines include many recommendations for the use of CMR which are briefly summarized in the next paragraphs and figures. This summary does not include non-CMR recommendations as the full guidelines are published in the *Journal of the American College of Cardiology* [1] and *Circulation* [2]. They are also planned to be published later this year in the *Journal of Cardiovascular Magnetic Resonance*. Reviewing the full Guidelines is necessary to get a detailed appreciation for how the various imaging modalities are ‘weighted’ in particular recommendations. Recommendation number from the full document is included for reference (Fig. 1).

### Low risk coronary artery disease patients

For low-risk coronary artery disease (CAD) patients, defined as those with a 30-day risk of death or major adverse cardiovascular event (MACE) < 1%, the 2021 AHA/ACC Chest Pain Guidelines provide a class 2a recommendation indicating that it is reasonable to discharge the patient home without hospitalization or urgent cardiac testing. This is one of the “Top 10 Take-Home Messages” of the updated guidelines.

Reducing imaging indications will reduce imaging costs and the number of cardiac tests that confirm no evidence of significant CAD. While an initial strategy of no-testing is appropriate in these patients, testing remains an option for patients with persistent or worsening symptoms. From a CMR perspective, there is time to schedule appropriate patients in a non-acute setting rather than doing imaging as an emergency procedure.

### Intermediate risk patients without known CAD

**Fig. 2:**
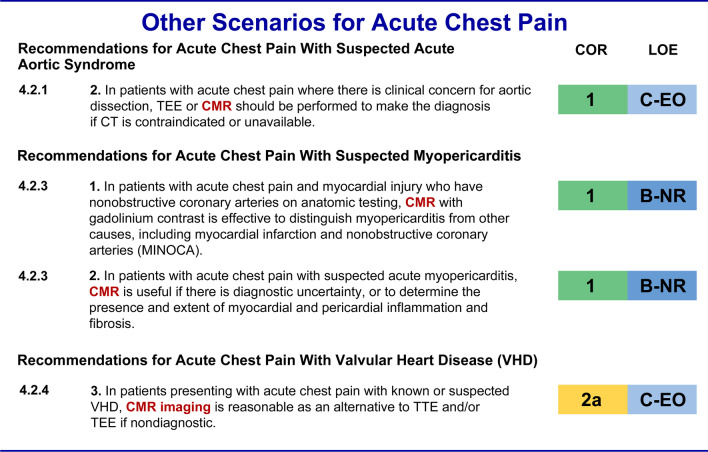
Other Scenarios for Acute Chest Pain—Recommendations for CMR [1, 2]. *CT* computed tomography, *MINOCA* myocardial infarction with no obstructive coronary arteries, *TEE* transesophageal echocardiography, *TTE* transthoracic echocardiography, *VHD* valvular heart disease. Other abbreviations as in Fig. 1

The 2021 AHA/ACC Chest Pain Guideline gives a class I indication for stress CMR along with all other stress imaging modalities among intermediate-risk patients *without* known CAD (4.1.2.1.4). Stress imaging, including CMR, is given a class 2a recommendation for sequential testing after an inconclusive coronary computed tomography angiographic (CCTA) study (4.1.2.1.7). The 2021 AHA/ACC Chest Pain Guideline puts CMR on par with the other stress imaging modalities regardless of whether or not a patient can exercise or whether or not the electrocardiogram (ECG) is interpretable (Fig. 2).

### Intermediate risk patients with known CAD

For intermediate risk patients with acute chest pain and *known* CAD, all stress imaging modalities are given a class 2a recommendation (4.1.2.2.5) along with CCTA, in patients with previously known non-obstructive CAD, and without any preference of one modality over the others. This recommendation also brings CMR to the same level as the other non-invasive imaging modalities. Of note, among those with known CAD, exercise testing *without* imaging is no longer considered an appropriate study.

### High-risk patients with acute chest pain

There is a class 2a recommendation for CMR or echocardiography to establish alternative diagnoses once obstructive CAD has been excluded by CCTA or invasive coronary angiography (ICA). CMR has become well established for detecting other pathologies such is myocardial infarction with no obstructive coronary arteries (MINOCA) (4.2.3.1) or myocarditis which can present acutely in the absence of obstructive CAD (4.1.3.3). Stress CMR could also be used to establish the diagnosis of ischemia in patients with no obstructive coronary arteries (INOCA) or microvascular disease (5.2.2.3).

The 2021 AHA/ACC Chest Pain Guidelines favor computed tomography (CT) as the first choice in the assessment of possible acute aortic syndromes given its faster speed and wider availability. CMR is given a class I recommendation as an alternative if CT is contraindicated or unavailable (4.2.1.2).

In patients with possible myopericarditis (4.2.3.2), the new guidelines give CMR a class I recommendation for distinguishing myocarditis from other causes and for assessing myocardial and pericardial inflammation and fibrosis. These recommendations align with increasing community awareness of CMR as the test of choice for an indication often labeled MINOCA. (4.2.3.1).

**Fig. 3:**
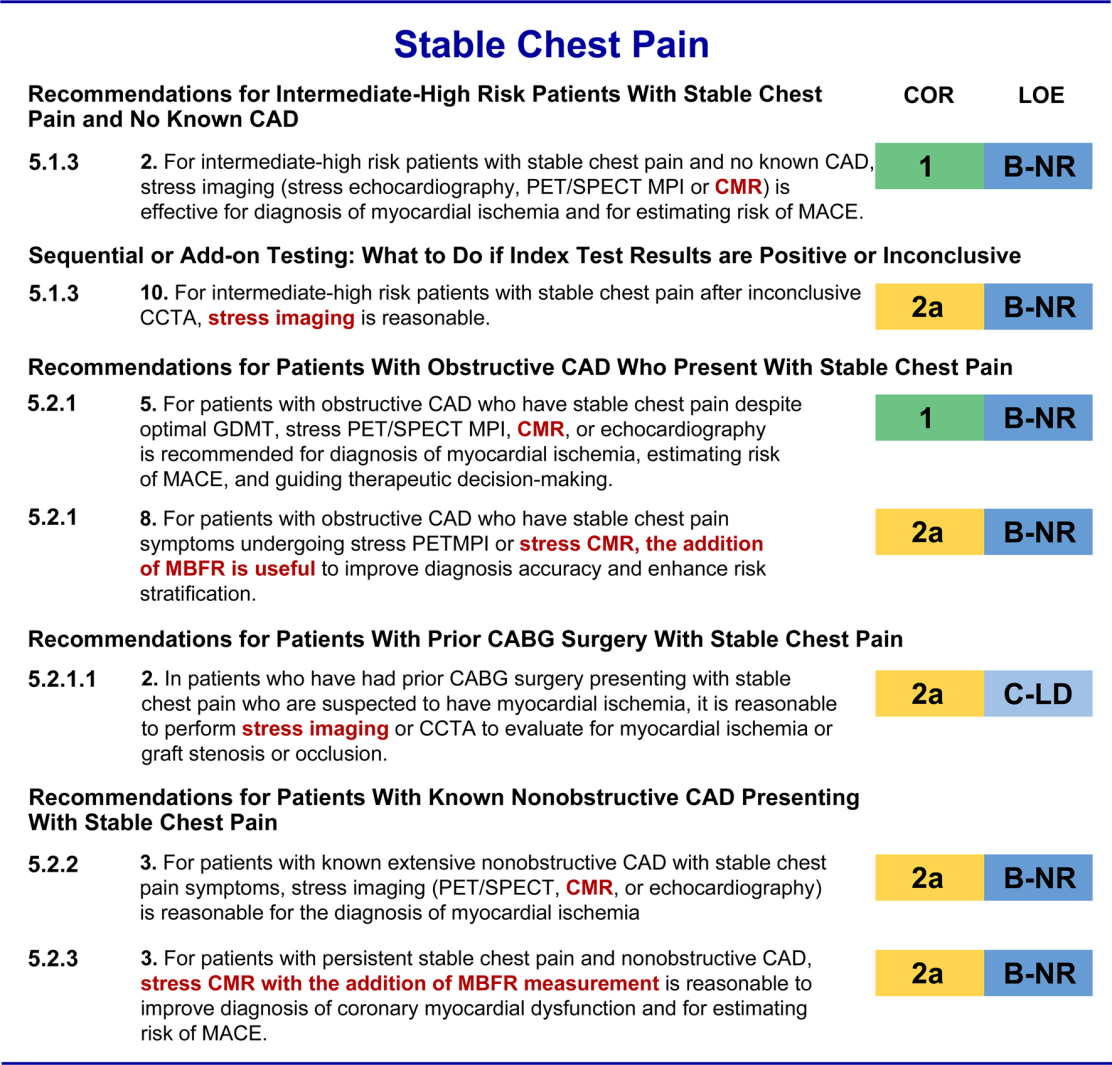
Stable Chest Pain Guidelines for CMR [1, 2]. INOCA, ischemia with no obstructive coronary arteries; MACE*,* major adverse cardiovascular event; *MBFR*, myocardial blood flow reserve. Other abbreviations as in Figs. 1 and 2

The 2021 AHA/ACC Chest Pain Guidelines also support a class 2a recommendation among patients with acute chest pain and known or suspected valvular heart disease if transthoracic echocardiography (TTE) or transesophageal echocardiography (TEE) are not technically adequate for assessing valvular heart disease (4.2.4.3). CMR has the ability to objectively quantify the severity of regurgitant heart lesions (Fig. 3).

### Intermediate-high risk patients with stable chest pain and no known CAD

Among intermediate-high risk patients with stable chest pain with no known CAD, CCTA and stress imaging are given class I recommendations (5.1.3.2). Again, CMR is not differentiated from the other stress imaging modalities.

### Stable patients with known CAD

In patients with known obstructive CAD who have stable chest pain despite optimal therapy, stress imaging receives a class I indication for diagnosing myocardial ischemia, estimating risk of MACE, and guiding therapeutic decision making (5.2.1.5). There is an additional new class 2a indication for both PET and CMR adding quantification of myocardial blood flow reserve (MBFR) to improve diagnostic accuracy and enhance risk stratification (5.2.1.8). This is particularly important given the growing availability of CMR techniques for performing absolute quantification of myocardial blood flow.

In patients with known non-obstructive CAD, stress CMR received a Class 2a recommendation for assessing INOCA (5.2.2.3). Stress CMR with the addition of quantitative myocardial blood flow assessment is given a class 2a recommendation for the diagnosis of coronary microvascular dysfunction and for assessing MACE (5.2.3.3).

## A case example from the meta-analysis literature (Knuuti et al. [4])

**Fig. 4:**
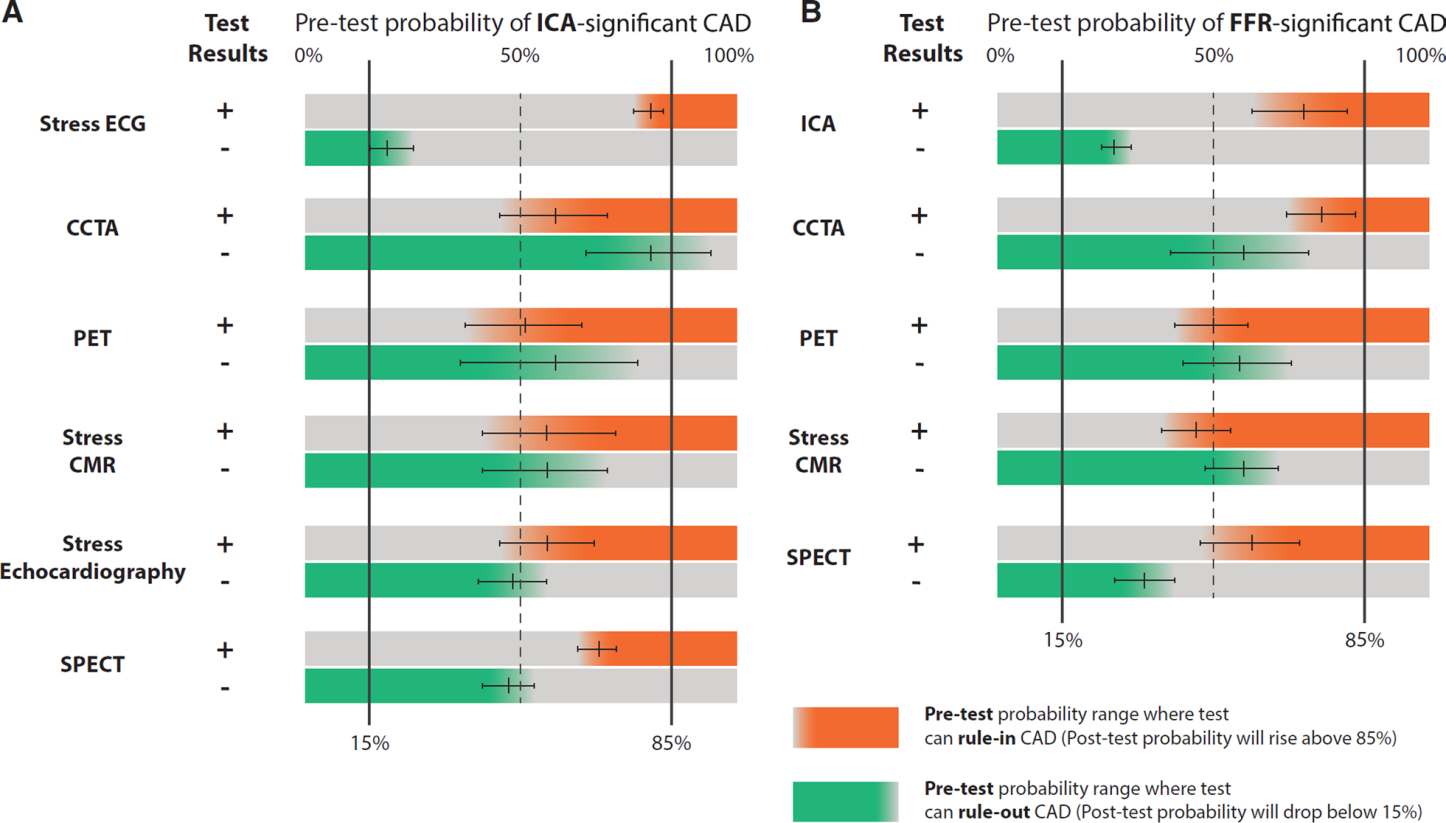
Ranges of clinical pre-test probability in which each single-positive test will confidently rule-in (in orange) the presence of significant CAD with post-test probability > 85% or, conversely a negative test will confidently rule-out CAD (in green) with post-test probability < 15% [4]

*“In a 55-year old male patient with atypical angina CCTA, single photon emission computed tomography (SPECT), PET and stress CMR can reliably rule-out anatomically significant CAD but stress ECG or stress echocardiography cannot (A). To assess the performance of imaging tests to detect functionally significant CAD (assessed by fractional flow reserve (FFR)) in the same patient (B) one can see that PET and stress CMR results can both rule-out and rule-in significant CAD while CCTA can only confidently rule-out if a negative result is documented. ICA and SPECT are not recommended tests in this patient.”* (Fig. 4)

## Disagreement with some aspects of the guidelines

### The recommendations of FFR-CT are premature

The CCTA field recognizes that CCTA (and ICA) cannot confidently rule-in functionally significant CAD and now advocates fractional flow reserve (FFR)-CT to determine functional significance of what is anatomically described as “obstructive CAD” (50–90% stenosis) and extending further to a 40% narrowing. A stress CMR may be more cost-effective than FFR-CT or PET in sequential testing. While, CCTA may be the best test to exclude anatomically significant CAD, PET and CMR can effectively rule-in and rule-out functionally significant CAD.

The 2021 AHA/ACC Chest Pain Guidelines have 4 recommendations for FFR-CT in acute and chronic stable chest pain syndromes and FFR-CT was broadly described as an instrument for assessing likelihood of ischemia with established robustness for decision-making in lesions of 40–90% detected on CTA. FFR-CT was displayed in numerous flow charts to represent equivalent class 2a recommendations compared to any other functional imaging modality.

However, the diagnostic and prognostic utilities of FFR-CT are not as robustly evidenced as any of the stress imaging modalities (stress CMR, SPECT, PET, and stress echocardiography). FFR-CT does not improve the sensitivity of CT, and only modestly improves the specificity of identifying flow-limiting obstructive coronary artery lesions when compared with invasive FFR and functional testing [5–8]. FFR-CT has only limited diagnostic accuracy in detecting hemodynamically significant CAD in the intermediate range of coronary stenosis of 0.6–0.85 where management decisions are most needed [7]. Studies evaluating FFR-CT have shown inferior incremental diagnostic and prognostic value in comparison to functional testing [7, 9]. Additionally, the recent FORECAST (Fractional Flow Reserve Derived From Computed Tomography Coronary Angiography in the Assessment and Management of Stable Chest Pain) trial of over 1400 patients, while referral to invasive angiography was lower, the use of FFR-CT did not demonstrate any benefits in terms of healthcare costs, cardiovascular outcomes, or quality of life compared to CT-alone [10]. Similarly, the recent RAPID-CT (Rapid Assessment of Potential Ischaemic Heart Disease with CTCA) trial [11] included 1748 patients with intermediate risk with suspected or a provisional diagnosis of acute coronary syndrome randomised to Early CCTA and standard of care compared with standard of care only. The study demonstrated that early CCTA did not alter overall coronary therapeutic interventions or one-year clinical outcomes.

Furthermore, there are practical implications regarding FFR-CT. FFR-CT is only feasible in a subset of CCTA cases that are relatively artifact-free and remains highly limited in patients with prior coronary artery stenting, extensive calcification, severe valvular heart disease, sequential luminal lesions or prior coronary artery bypass graft surgery. FFR-CT is currently only available by a single company (HEARTFlow, Redwood City, California, USA). The CPT Category III code used to reimburse FFR-CT is reserved for emerging technologies. Finally, FFR-CT costs 3-times as much as a standard CCTA.

### The ischemia imaging modalities were inappropriately compared as a group against anatomical CCTA

The 2021 AHA/ACC Chest Pain Guidelines put all stress imaging modalities in the same “functional imaging” group for the purpose of comparison against CCTA, as shown in many flow charts and tables. This approach has little clinical basis but also misses critical attributes of different ischemia tests that may be relevant in the management of a vastly diverse patient spectrum. In the contemporary era, there is clear randomised trial evidence for the use of stress CMR in reducing unnecessary ICA referral or coronary revascularization rates, and thus improving patient care, health outcomes and healthcare resource utilization [12, 13].

The largest randomized trial to date, PROMISE (PROspective Multicenter Imaging Study for Evaluation of chest pain), prospectively evaluated the utility of an anatomic (CCTA) testing approach in comparison with functional testing (stress imaging and treadmill ECG testing) amongst 193 North American centers in over 10,000 patients [14]. After a median of 2-years follow-up, no differences in adverse cardiac outcomes were observed, while the anatomic approach led to higher downstream utilization of both ICA and coronary revascularization.

The SCOT-HEART (Scottish COmputed Tomography of the HEART) trial conducted within the United Kingdom failed to meet its original primary endpoint, but did observe a late reduction in non-fatal myhocardial infarction (MI) [15]. However, it was a trial of serial testing in one arm (standard care using ETT plus CCTA) versus standard of care (ETT only). This form of layered testing might be expected to produce better outcomes, especially as exercise tolerance testing (ETT) without imaging is well recognized for lower sensitivity and specificity than stress imaging tests. In addition, participants in the SCOT-Heart trial were not systematically treated with optimal medical therapy for primary prevention, a standard of care that has been part of modern cardiology practice for many years.

### Definitions of obstructive CAD, anatomically significant CAD, and functionally significant CAD

The 2021 AHA/ACC Chest Pain Guidelines do not have clear definitions of “Obstructive CAD”, “Anatomically Significant CAD”, and “Functionally Significant CAD”. These three definitions overlap with each other and contribute to miscommunication among healthcare providers and confuse patients. In general, the 2021 AHA/ACC Chest Pain Guidelines do not systematically differentiate anatomic CAD and functional CAD. This may contribute to an overestimation of the utility of CCTA compared with stress imaging modalities.

“Obstructive CAD” has been used to describe a 50% or greater coronary artery diameter stenosis by quantitative coronary angiography (QCA) and is now widely used to describe CCTA findings. However, the majority of 50–70% stenoses are not severe enough to impair coronary flow reserve [16]. *Invasive* FFR has been shown in several multi-center trials to provide better outcomes than management by stenosis severity [17–19]. Thus, “functionally significant CAD” represents the subset of coronary artery stenoses that impair flow during vasodilation or increased coronary flow demand. Stress imaging tests are inherently designed to detect abnormal flow reserve detected during exercise or pharmacological stress. We believe the cardiology community should work to clarifying terminology and avoid using words like “obstructive” that suggest a physiological importance to an anatomic stenosis that may or may not impair coronary flow reserve.

### Women’s health

The 2021 AHA/ACC Chest Pain Guidelines briefly mention women-specific considerations in text but do not make any formal recommendations that recognize appropriateness or level of evidence. CCTA, SPECT, and PET directly deliver radiation to the breasts. Now that zero ionizing radiation methodologies like CMR can perform as well as CCTA and PET, and are superior to stress echocardiography and SPECT, CMR seems the logical choice for stress perfusion imaging in women if local equipment and expertise is available.

## Concluding thoughts and directions for future research

**Fig. 5:**
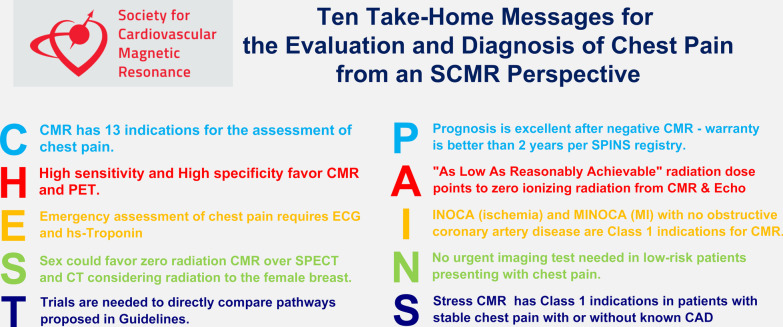
Ten Take-Home Messages for the Evaluation and Diagnosis of Chest Pain from an SCMR Perspective

CMR has matured into a powerful diagnostic tool as evidenced by the wide range of clinical indications recognized in the 2021 AHA/ACC Chest Pain Guidelines and other international guidelines. Figure 5 summarizes 10 Take-Home Messages for the Assessment and Diagnosis of Chest Pain—from a SCMR perspective. CMR practitioners should continue to advocate the importance of the functional significance of CAD.

The CMR community should be proud of the hard work that provided the data leading to multiple Class I and Class 2a Recommendations, which are now finally more aligned with the 2014 ESC Guidelines on Revascularization in which stress CMR features in Class IA recommendations [20]. However, more research is needed regarding the comparative effectiveness of CMR relative to other stress imaging techniques and specifically, CCTA. Randomized clinical trials comparing costs and outcomes of different management strategies will be important.

CMR and PET appear to have superior diagnostic accuracy compared with SPECT and stress echocardiography. The relatively low specificity of CCTA for functionally significant CAD is a weakness. The reliance on FFR-CT to provide a computer-based substitute for a full physiological assessment of CAD may or may not be cost-effective over the long haul. CMR researchers should also continue to refine quantitative methods as the current Guidelines are the first to formally recognize the value of quantifying myocardial blood flow reserve by CMR. Studies focused on women will help highlight the role of stress CMR versus other modalities in the diagnosis of CAD as well as other causes of symptoms.

## Data Availability

All data and materials are in the public domain and thus available.

## Abbreviations

ACC: American College of Cardiology
AHA: American Heart Association
CAD: Coronary artery disease
CCTA: Coronary computed tomography angiography
CMR: Cardiovascular magnetic resonance
CT: Computed tomography
ECG: Electrocardiogram
ETT: Exercise tolerance test
FFR: Fractional flow reserve
ICA: Invasive coronary angiography
INOCA: Ischemia with no obstructive coronary arteries
MACE: Major adverse cardiovascular event
MBFR: Myocardial blood flow reserve
MI: Myocardial infarction
MINOCA: Myocardial infarction with no obstructive coronary arteries
MPI: Myocardial perfusion imaging
PET: Positron emission tomography
QCA: Quantitative coronary angiography
SCMR: Society for Cardiovascular Magnetic Resonance
SPECT: Single photon emission computed tomography
TEE: Transesophageal echocardiography
TTE: Transthoracic echocardiography
VHD: Valvular heart disease
